# Systematization of Nursing Care: seeking defining and differentiating theoretical contours

**DOI:** 10.1590/1980-220X-REEUSP-2021-0504

**Published:** 2022-03-30

**Authors:** George Luiz Alves Santos, Glaucia Valente Valadares

**Affiliations:** 1 Universidade Federal do Rio de Janeiro, Escola de Enfermagem Anna Nery, Rio de Janeiro, RJ, Brazil.

**Keywords:** Nursing Process, Nursing Services, Health Management, Workforce, Organization and Administration, Professional Practice, Proceso de Enfermería, Servicios de Enfermería, Gestión en Salud, Recursos Humanos, Organización y Administración, Práctica Profesional, Processo de Enfermagem, Serviços de Enfermagem, Gestão em Saúde, Recursos Humanos, Organização e Administração, Prática Profissional

## Abstract

The objective of this study was to elaborate theoretical propositions to help understanding the Systematization of Nursing Care as a distinct phenomenon of the Nursing Process. It is a reflective, theoretical study that presented two categories: “Systematization of Nursing Care: seeking differentiating contours” and “Systematization of Nursing Care: seeking defining contours”. It was identified that the systematization is not concerned with guiding the professional nursing care itself, since this issue, together with the guidelines regarding the elaboration of clinical documentation resulting from the implemented nursing care, is related to the process. Despite what the Systematization of Nursing Care is, the idea is that it is a field of knowledge that, through its three pillars and constituent elements, supports the structuring of nursing services and, consequently, the organization or reorganization of professional work in nursing. It is concluded that systematization, in the light of the theoretical propositions presented here, is an area of knowledge that represents a particular way of carrying out the management of nursing services when the three pillars that compose it are considered.

## RESUMO

Objetivou-se elaborar proposições teóricas que auxiliem na compreensão da Sistematização da Assistência de Enfermagem enquanto fenômeno distinto do Processo de Enfermagem. Estudo teórico reflexivo que apresentou duas categorias: “Sistematização da Assistência de Enfermagem: buscando contornos diferenciadores” e “Sistematização da Assistência de Enfermagem: buscando contornos definitórios”. Identificou-se que a sistematização não se ocupa em orientar a assistência profissional de enfermagem em si, já que essa questão, aliada às orientações quanto à elaboração da documentação clínica resultante da assistência de enfermagem implementada, diz respeito ao processo. A despeito do que é a Sistematização da Assistência de Enfermagem, tem-se que se trata de um campo de conhecimento que, por meio de seus três pilares e elementos constituintes, suporta a estruturação de serviços de enfermagem e, por consequência, a organização ou reorganização do trabalho profissional em enfermagem. Conclui-se que a sistematização, à luz das proposições teóricas aqui apresentadas, constitui área de conhecimento que representa uma forma particular de realizar a gestão de serviços de enfermagem quando se consideram os três pilares que a compõem.

## RESUMEN

Se propuso elaborar proposiciones teóricas que auxilien en la comprensión de la Sistematización de la Asistencia de Enfermería como un fenómeno distinto del Proceso de Enfermería. Estudio teórico reflexivo que presentó dos clases: “Sistematización de la Asistencia de Enfermería: buscando contornos diferenciadores” y “Sistematización de la Asistencia de Enfermería: buscando contornos definitorios”. Se identificó que la sistematización no se ocupa en orientar la asistencia profesional de enfermería por si misma, una vez que esa cuestión, sumada a las orientaciones a respecto de la elaboración de la documentación clínica resultante de la asistencia de enfermería implementada, se refiere al proceso. A pesar de lo que es la Sistematización de la Asistencia de Enfermería, se conviene definir como un campo de conocimiento que, por medio de sus tres pilares y elementos que la constituyen, soporta la estructuración de servicios de enfermería y, por así decirlo, la organización o reorganización del trabajo profesional en enfermería. Se concluye que la sistematización, siguiendo las proposiciones teóricas presentadas, constituye área de conocimiento que representa una forma particular de realizar la gestión de servicios de enfermería cuando se consideran los tres pilares que la componen.

## INTRODUCTION

Understanding the organization of professional work^(1)^, how it is processed in the practice of Brazilian nursing services, what theoretical bases support it and who the agents responsible for it are, when care activities of nurses, midwives, nursing technicians, nursing assistants are considered, is a theoretical and practical challenge that still lacks disciplinary reflections. Professional work organization requires considering, according to Resolution 358 of 2009 of the Federal Council of Nursing (*Cofen*)^(1)^, the method, personnel, and instruments, fundamental pillars of the Systematization of Nursing Care (*SAE*).

The literature has pointed out that the work organization is related to the “process involving the workers’ activities, the work relations with their peers and with the hierarchy, and that takes place in a certain institutional structure”^(2)^. When taking this perspective in consideration, look goes beyond care, encompassing issues that are greater than care itself, contemplating the nursing service by placing it in an organizational perspective in a given health service. The same authors cite that such an organization “suffers structural influences, related to the macroeconomic structure, as well as organizational ones, such as the management mode undertaken by the institution, which is, in its turn, related to the current mode of production”^(2)^. Based on this perspective, the organization of professional work^(1)^ holds predicates related to nursing management in health services.

*Cofen* has made efforts over the years to ensure the necessary conditions regarding the implementation of the Nursing Process (NP) in the various care contexts that demand professional nursing care. This operationalization would be possible through *SAE*. However, what seems easy to understand, that is, that SAE organizes professional work^(1)^, still raises wide-ranging discussion about what such an organization would mean, since important gaps have persisted since 2002 with the approval of the first resolution^(3)^ dealing with the subject.

Resolution 272^(3)^, at that time, addressed SAE and NP in its text as similar phenomena and, 18 years later, still evokes reflections that would contribute to the clear delimitation of the conceptual and operational differences about the terms, as well as the *modus operandi* of SAE in the organization of professional work^(1)^. Undoing the terminological and conceptual construction and also delimiting the predicates that allow the apprehension of the phenomenal identity of SAE as distinct from the NP is necessary for advances in disciplinary knowledge. SAE is essentially involved in the management of nursing services, when it proposes the organization of professional work. NP, in its turn, is involved in guiding care and documenting practice^(1)^. However, even though the current resolution has advanced in pointing out the purpose of each term, it has not conceptually and operationally defined SAE, its three pillars, nor the elements that make up each of them.

In the same resolution^(3)^, SAE and NP were identified as similar steps. The first resolution, although revoked, still features the Brazilian professional discourse significantly and contributes to overshadowing, at times, the corrections that resolution 358 of 2009 made. Nevertheless, among professionals and even in the national scientific nursing production, expressions such as “implementing SAE” or “undertaking SAE” are recurrent. In addition, “register SAE” or “do SAE” are common findings. This perspective brings SAE closer to the main function of NP, which is to guide professional care and practice documentation^(1)^ and contributes to the mistaken understanding that such terms mean the same phenomenon.

It is understood as pertinent that expressions such as “implement” or “undertake” SAE are not the most appropriate when considering the diagnostic analyses regarding the presence or absence of its pillars and the constituent elements that compose them. It is suggested to consider, for example, that, when a new nursing service is inaugurated, what best characterizes this moment would be the action of organizing the professional nursing work mentioned in Resolution 358^(1)^. In its turn, when a given service, on its own initiative, impelled by the supervision of the Regional Nursing Councils, or even by spontaneous institutional adhesion to a given accreditation seal, makes institutional changes, the action that best characterizes that moment would be to (re)organize the nursing work.

The nursing literature has identified schools of thought that corroborate the issue raised here. Three schools of thought were identified, namely: the first school treats SAE, NP and nursing care methodology (*MAE*) as distinct terms; a second school considers NP and *MAE* synonyms; and a third school considers SAE, NP, and MAE synonyms^(4)^. Conceptual confusion and doubt regarding the use of SAE, NP and taxonomies have still been pointed out in the nursing literature^(5)^. The taxonomies are exemplified by the one from NANDA-International (NANDA-I) Nursing Diagnoses^(6)^, the Classification of Nursing Outcomes^(7)^, Classification of Nursing Interventions^(8)^ and International Classification for Nursing Practice (ICNP^®^)^(9)^.

An editorial^(5)^ discussing about SAE brought the provocation that it is a Brazilian invention and questions: “Why the creation of SAE in Brazil, if the Nursing Process is itself the model of professional practice? Why, in Brazil, does SAE stand out to the detriment of the Nursing Process?” These are issues that deserve reflection, as it is advocated that SAE and NP are distinct terms/concepts and coined with different purposes, although they are (inter)related in the daily life of nursing services and have a convergent purpose, that is, on one hand, to qualify the nursing service management through SAE and, on the other hand, to qualify the professional practice of Brazilian nurses through the NP.

The term SAE has been used nationally to refer to the organization of care through the use of the phases of the scientific method; however, this association may not suit the phenomenon in question, since systematizing can mean organizing, but necessarily using all the stages of such a method^(10)^. Thus, it still seems pertinent to question in contemporary times: is there agreement on the concept of SAE?^(11)^.

A standard edited by Cofen points out that SAE aims to allow NP operationalization^(1)^. In this regard, it is understood that it has the indispensable conditions for the implementation and undertaking of the NP. The differentiation between the terms would be better understood, then, by its purpose, that is, the “for what”, and, thus, SAE would organize the professional work for the operationalization of the NP^(1)^. However, there is still a lack of theorization to anchor the ontology of what SAE is, elucidating what it is, or at least presenting clear epistemological contours.

A critical point is to consider that SAE would operationalize something that, in theory, makes up one of its own pillars, since the NP is directly related to the method pillar, being indicated as one of its elements^(12)^. The literature^(5)^ states that the pillar method could be understood as the NP itself, and, in this way, would be contained in SAE, whose questioning about the issue is: Would the NP be something smaller, or even an axis of SAE? It is problematized that SAE exists as a function of the NP and, therefore, an effort is required to understand the place of NP in this context, as it should not be considered an integral part of the tool that gathers the conditions for it to be effective in everyday professional practice.

Revisiting the current resolution, the term “Nursing Process” is considered to have clearer epistemological contours than those of SAE, allowing the comprehension of what NP is and how it is operationalized. Moreover, its five stages are already well defined, in which data collection, diagnosis, planning, implementation, and nursing evaluation^(1)^ already have well-defined conceptual and operational aspects. This way, it is possible to clearly understand what NP is and how it is operationalized in practice, as well as its context of use – nursing care, the genesis of its implication. Whether the time has come for Brazilian nursing, through its competent bodies, review and update Resolution 358 of 2009 is also a question.

Taking in consideration the ethical aspects making a criticism and reaffirming the full respect for Brazilian production on the subject, since there are three schools of thought circulating^(4)^, terminological and conceptual mistakes are common when accessing the production on SAE. Not infrequently, studies approach the theme of SAE and, in whole or in part, these studies are based on the NP, contributing to the maintenance of mistaken uses of such terms. Therefore, understanding SAE conceptually and operationally can help settle or at least minimize such misconceptions.

In view of the above, the guiding question of this research is: what theoretical propositions contribute to the delimitation of defining epistemological contours of SAE? The objective of this study was to elaborate theoretical propositions that help in the understanding of SAE as a distinct phenomenon from NP.

## METHOD

Reflective theoretical study supported by Cofen resolutions that deal with the theme and in a *corpus* of selected national texts. The selection of Brazilian texts was privileged because SAE is a common theme for national nursing. This study is also part of the reflections that the first author experienced when entering the field for data collection, considering the thesis in preparation, entitled “Meanings attributed to SAE: implications for health care”.

### Systematization of Nursing Care: Seeking Differentiating Contours

This category performs the reflexive exercise of presenting theoretical propositions that help to differentiate SAE from NP, as distinct phenomena that permeate the spaces of care production. First, what the SAE is not is outlined, so that, in a second moment, it is possible to (re)construct the understanding of this phenomenon and, then, point out theoretical clues to indicate what SAE would be.

Aspects related to NP shall be made explicit so that, from them, it is possible to better understand the epistemological contours of SAE. NP takes place in a procedural way in a *continuum* of a care situation, in which this would be “the way of doing contemporary nursing work, the way of scientific use of nursing”^(5)^, consisting of five stages, phases, and components^(1)^, of which the nursing diagnoses, outcomes, and interventions “constitute a way of doing and a way of thinking of nursing practice”^(13)^.

The implementation of the NP shall be anchored in a nursing theory that supports the procedural and cyclical movement that is characteristic of the NP and directs its implementation, maximizing its therapeutic purpose, which is to guide professional care and documentation of practice^(1)^. One of the unequivocal objectives of the NP, considering its specific phases, lies in the identification, naming, establishment of outcomes, and the treatment of a “human response to health conditions/life processes, or to a vulnerability to this response”^(6)^ by the clients under the clinical responsibility of the nurse and his/her team.

It should be emphasized that there is a need to consider that nursing theories guide the application of their elements to professional practice through the NP^(13)^. As an example, to anchor the NP, we have the Interactive Theory of Breastfeeding that: “describes, explains, predicts and prescribes breastfeeding, examining the preceding and influencing factors, as well as the consequences in the breastfeeding process”^(14)^.

In addition to nursing theories, professional practice through NP can be anchored in other theoretical frameworks. In this regard, we mention the study that aimed to identify which theoretical frameworks guide nursing clinical practice in mental health, identifying that, besides nursing theories, the “biomedical framework”, frameworks supporting alternative practices, framework supported by the public policy of harm reduction, and framework of psychoanalytic theory support nursing practice”^(15)^.

NP can be understood as the “main methodological model for the systematic performance of professional practice, or a technological instrument used to favor care”^(16)^. This way, the direct implication of NP on the professional nursing care at the bedside is outlined, characterized mainly by care actions directly linked to the patient. This aspect is essential to understand the distinction between the terms, because from the understanding of what NP is, one can theorize what does not characterize SAE, differentiating it from NP.

**Chart 1.**
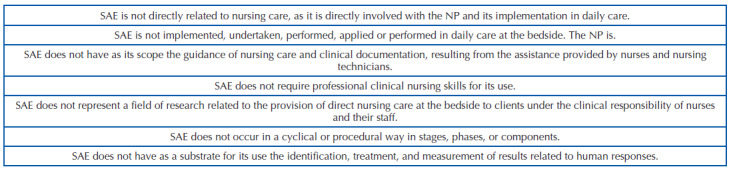
Theoretical propositions that delimit what the Systematization of Nursing Care is not.

SAE: Systematization of Nursing Care; NP: Nursing Process.

To better assist in understanding what SAE is not, theoretical propositions were established from the reflections that guided this text (Chart 1). It should be noted that these propositions shall be subjected to scientific scrutiny in the future, so that they can be validated or refuted, considering the advance in the production of knowledge on the subject. However, they can be considered, so far, advances that go beyond what has been produced about SAE, due to the purposeful action of differentiation that they allow taking place.

In summary, SAE constitutes an epistemological object distinct from NP and is not NP itself. It does not deal with direct assistance phenomena and human responses that are the genesis of the NP’s implication. Rather, based on its three pillars, it gathers the indispensable conditions for organizing professional nursing work^(1)^, to accomplish the operationalization of NP in daily professional practice in the different care contexts.

### Systematization of Nursing Care: Seeking Defining Contours

This category of analysis performs the exercise of clearly delimiting that SAE shall approach studies that deal with research objects related to the organization of professional nursing work^(1)^. Care must also be taken not to understand SAE as a substitutive knowledge regarding nursing administration. This is because SAE is not nursing management or administration, but represents a peculiar field of knowledge production, which is closely related to such areas. It can be said that SAE is a customization in the way of organizing nursing services and managing them.

The nursing literature has pointed out that SAE supports nursing management^(5)^. However, we disagree with this perspective, since SAE is anchored in administration principles; therefore, knowledge in nursing management supports SAE that, consequently, allows the organization of professional work^(1)^ – and not the other way around. SAE is a customized tool that Brazilian nursing coined and standardized to characterize the administrative practice necessary for the operationalization of NP through the organization of nursing professional work^(1)^.

Considering the advances and setbacks associated with SAE, it is considered that the first ones appeared as a resolution was enacted legally requiring nursing services to have minimum standards of organization of professional work, so that it is possible to operationalize NP^(1)^. This can be considered an advance. As setbacks, it can be said that the absence of consensus regarding conceptual and operational understanding, as well as in terms of propositions, considering SAE phenomenon, can minimize the real contributions it has to nursing and health services. Consequently, the real contribution of NP to professional practice can be minimized, since such issues are not commonly discussed.

Resolution 358 of 2009^(1)^ does not define, conceptually nor operationally, what SAE is, the method, personnel and instruments. This text covers that method, personnel, and instruments need to be understood in a different perspective from the stages or phases of NP^(1)^. Thus, they do not have a procedural and cyclical character. In the nursing literature, they are identified as axes^(5)^, but it seems that this term cannot translate the structural idea that will be further presented. Reflecting on the possibilities of representation of the three terms, it is understood that expressions such as mainstay, framework, column, support, base, and pillar^(17)^ can better represent them.

The word pillar, according to the dictionary concept, can be understood as a “simple column that supports a construction”^(17)^. This seems suitable to be used and to represent the structure or anatomy of SAE, based on the method, personnel, and instruments. Thus, it is inferred that the three pillars of SAE would comprise domains of professional knowledge essential to the organization of professional work mentioned in Resolution 358^(1)^, but possibly do not cover the full range of knowledge necessary for such activity.

**Figure 1.**
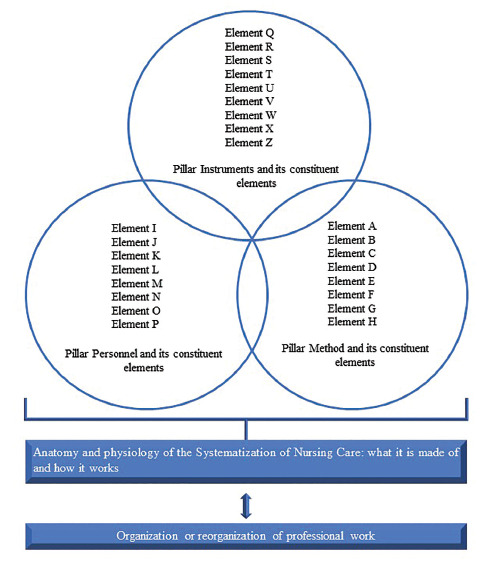
Anatomy and physiology of the Systematization of Nursing Care.

Source: Adapted from Hulley et al.^(18)^.

Each pillar would serve as a structural framework for SAE. However, it should be borne in mind that each pillar is made up of smaller parts, that is, basic elements that shape them as structuring pillars. Based on Hulley et al.^(18)^, the understanding of SAE is proposed originated from its anatomy (what it is made of), as well as from its physiology (how it works). In Figure 1, an explanatory scheme can be seen that includes the three pillars and the constituent elements of SAE in an anatomical and physiological perspective.

When such pillars and their constituent elements are present in a given care context – a nursing service, it is understood that the anatomy of SAE is present and, thus, there are the necessary conditions known so far for the operationalization of the NP. However, such pillars and constituent elements have to be interconnected, in harmony with NP so that the use of each constituent element by nurses is a possible daily – operational reality. The points of intersection between the circles mean that the pillars and constituent elements are present but are also harmonious with each other and functioning, thus providing opportunities for professional practice from the NP and the functioning of the nursing service in question – revealing the physiology of SAE.

Returning to the anatomy of SAE, it consists of three pillars. The pillar method can be understood from the following constituent elements: nursing theories and NP/nursing consultation. NP is related to nursing taxonomies and nursing records^(12)^. Nursing anamnesis and physical examination are also considered related to the method as subsidies for the implementation of NP. The other theoretical references involved in clinical nursing practice and, thus, assumed to have an intrinsic relationship with the method pillar should also be mentioned.

The pillar personnel has been associated with team sizing^(12)^. However, this perspective shall be extrapolated and encompass the nursing team, people management, and human resources training^(19)^. The pillar instruments has been associated with protocols, manuals, and printed material^(12)^. The nursing literature has offered more details of the constituent elements of the pillar instruments and indicates them as its members: “nursing manual, nursing service internal regulations, standard operating protocols, care protocols and bundles, clinical assessment scales, and nursing forms/printed material”^(19)^.

Another sensitive point is the production of knowledge about SAE in national journals. The existent limits and possibilities regarding SAE as an object of study that provokes questioning and, consequently, the elaboration of research problems to be solved by those who focus on the theme to produce science shall be identified. What is the standpoint theory for productions that deal with SAE? It is advocated that authors should, in their texts, among other aspects, make the theoretical position adopted clear regarding the term, so that knowledge can be produced and consumed, having clarity under which schools of thought^(4)^ discussions are settled.

**Chart 2.**
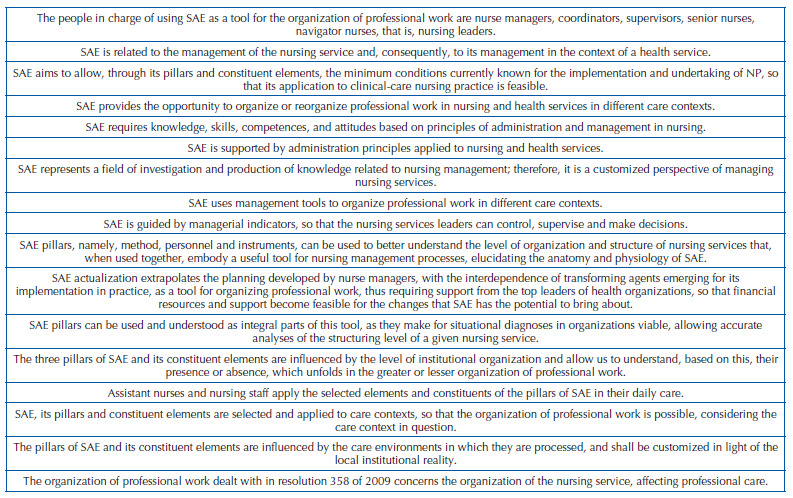
Theoretical propositions delimiting what the Systematization of Nursing Care is.

SAE: Systematization of Nursing Care; NP: Nursing Process.

SAE is a term that deserves special attention on the part of Brazilian nursing, since the terminological mistakes related to it, whether in care environments, in formal teaching spaces or even among researchers, have caused losses in the production of knowledge and in the epistemological delimitation when considering the term. Thus, theoretical propositions were elaborated, based on the reflections given here, aiming to broaden the understanding of SAE (Chart 2).

SAE, in the light of Resolution 358 of 2009, organizes professional work in terms of method, personnel and instruments, so that NP operationalization is possible^(1)^. However, the following provocation arises: would it operationalize only NP? The organization of professional work is accordant with the perspective of the work process organization or reorganization, understood as “organization and distribution of work activities in health services”^(20)^.

However, we should consider that the complexity of the nurse’s work process goes beyond the simple organization and distribution of activities^(20)^, since the literature has pointed out other work processes in nursing, namely the work process of assisting, managing, teaching, researching and participating politically^(21)^. This is of fundamental importance, because as already mentioned, there is possibly still a need to identify other pillars besides the existing ones that cover the range of work processes that involve the organization of professional nursing work.

## FINAL CONSIDERATIONS

This reflection reached the objective proposed, which was to present theoretical propositions that allowed to better understand and differentiate SAE from NP, even if incipiently and in the context of a theoretical reflection, which constitutes a limitation of the study. Therefore, SAE is not the NP and should not be approached as similar to it; it does not occur in stages and is not directly related to bedside care, although it has important implications, as it enables, through its three pillars, the operationalization of NP and, consequently, its implementation and undertaking.

Briefly, SAE does not configure an epistemological object that is directly related to bedside care, which is a privileged space for the implementation of NP – a guiding instrument for the care provided by nurses and nursing teams, which results in a series of clinical information, which need to be recorded and formalized through written communication.

Despite what SAE is, the term brings together predicates that characterize it as a field of knowledge related to the management and/or administration of nursing services. It cannot be said that SAE is nursing management, but it is understood that, through its pillars, it contributes to the organization of professional work in different care contexts, unfolding into a tool that can support the practice of nursing leadership management.

## ASSOCIATE EDITOR

Marcia Regina Cubas
